# PROMOTING EFFECTIVE DIAGNOSIS: THE PUBLIC HEALTH CENTER OF EXCELLENCE ON EARLY DETECTION OF DEMENTIA

**DOI:** 10.1093/geroni/igac059.276

**Published:** 2022-12-20

**Authors:** Joshua Chodosh

**Affiliations:** New York University Grossman School of Medicine, New York City, New York, United States

## Abstract

The Public Health Center of Excellence on Early Detection of Dementia brings together a broad coalition of stakeholders to assure widespread awareness of why early detection of dementia matters. The Center assesses and disseminates the best evidence supporting early detection and how to accomplish it – the first step on the pathway to high quality, effective diagnosis and health care for people living with dementia. The Center’s advisory group represents key state and county Departments of Health, healthcare systems and primary care providers, community organizations, and professional societies. The Center identifies effective materials and marketing strategies for increasing awareness about cognitive impairment, the value of earlier detection, and how best to conduct detection strategies. The Center works closely with state, county, and tribal public health agencies in dissemination, and seeks to establish collaborative linkages with healthcare systems and their primary care providers to build strategies for post-detection diagnosis and care.

